# The Impact of the COVID-19 Lockdown on European Students’ Negative Emotional Symptoms: A Systematic Review and Meta-Analysis

**DOI:** 10.3390/bs12010003

**Published:** 2021-12-23

**Authors:** Patrick Oliveira Carvalho, Thorben Hülsdünker, Fraser Carson

**Affiliations:** 1 Department of Sport and Exercise Science, LUNEX International University of Health, Exercise and Sports, L-4671 Differdange, Luxembourg; oliveira_carvalho.patrick@stud.lunex-university.net (P.O.C.); thorben.huelsduenker@lunex-university.net (T.H.); 2 Luxembourg Health and Sport Sciences Research Institute A.S.B.L., L-4671 Differdange, Luxembourg

**Keywords:** isolation, emotion, university, coronavirus, depression, anxiety, stress

## Abstract

Considerable changes to higher education approaches, as a response to the global coronavirus pandemic, has increased the stress on university students. The impact of these changes has had an effect on the negative emotional symptoms being experienced, which can lead to more severe mental health issues. The purpose of this meta-analysis was to determine the prevalence of anxiety, depression and stress during the coronavirus lockdown. A systematic review of three electronic databases (Google Scholar, PubMed and Medline) was conducted, with 13 studies from different European countries reporting data on students and their negative emotional symptoms identified. The random-effects model was used to perform the meta-analysis on anxiety, depression and stress. The overall pooled prevalence rate was 55% (95% CI: 45–64%) for anxiety, 63% (95% CI: 52–73%) for depression and 62% (95% CI: 43–79%) for stress. The impact of the coronavirus pandemic on negative emotional symptoms has been serious with studies reporting high prevalence rates for these. Isolation, reduced social contact, duration of quarantine and restrictions, which are the characteristics of a lockdown, played an important role in increased negative emotional symptoms for students. Countries have to be aware of this situation and develop mental support strategies to mitigate the impact.

## 1. Introduction

On 31 December 2019, Wuhan, China, reported the outbreak of the coronavirus disease 19 (COVID-19). One month later (30 January 2020), the World Health Organization (WHO) declared a Public Health emergency of international concern, followed by the confirmation of a global pandemic on 11 March 2020 [1]. This situation brought uncertainty to people and had a dramatic impact on the population. To slow down the propagation of COVID-19, countries were forced into lockdowns by isolating people in their homes, forcing companies, schools and universities to adopt a home-office and home-schooling strategy, the effect of which led to increases in negative emotional symptoms [2], with young adults between 18 and 24 years of age being one of the most affected groups [3].

Negative emotional symptoms are experienced as a response to difficult life circumstances, with the most commonly reported as anxiety, depression and stress [4]. An inability to manage these symptoms effectively can increase the potential for experiencing mental health issues [5]. The two continua model of mental illness and health [6] allows for mental health and mental illness to be separate, but related, constructs. In this way, an individual may experience high levels of anxiety, depression or stress without experiencing mental illness. Long periods without social interaction, as caused by quarantine, is known to increase stress levels and lead to poorer mental health [7]. During quarantine, stress increases as a result of frustration and boredom, inadequate supplies and inadequate information [8]. The impact of this is experiencing higher levels of anxiety and stress [8].

In normal circumstances, university students are exposed to numerous stressors, such as grade requirements, exams or volume of learning material [9]. Beiter [9] further recognized increased stress for students to be caused by the transition of leaving home and living alone (or with other unfamiliar people), making them more susceptible to experiencing negative emotional symptoms than the general population [10]. This exposes university students to a greater risk of developing mental health issues, which is augmented by the COVID-19 pandemic. At this time, many universities moved to online teaching formats, while canceling practical activities, which meant considerable changes to the studying environment for students [11]. The mass changes and uncertainty, coupled with a lack of opportunity to participate in social activities and engage in structured physical activity, left students highly susceptible to developing negative emotional symptoms [12].

The number of studies on the impact of COVID-19 is increasing with a major focus on general population and healthcare workers, who find themselves in the frontline of this battle against the coronavirus. The prevalence of negative emotional symptoms has seen a significant increase in 2021 with reports seven times higher than pre-pandemic levels [13]. For university students, the perceived stress and increased sedentary behaviour from COVID-19 has been reported to have had a significant effect on negative emotional symptoms [14]. There are numerous studies on this but also a lot of heterogeneity in methodological approaches as such, this review and meta-analysis aimed to analyze the impact of the pandemic on negative emotional symptoms in European university students during the lockdown period. The intent is to remove bias from the published literature and systematically quantify the overall Europe-wide effect of the COVID-19 pandemic on negative emotional states, which can allow for more appropriate intervention strategies to be implemented.

## 2. Materials and Methods

### 2.1. Search Strategy

A systematic search of three electronic databases (Google Scholar, PubMed and Medline) following the Preferred Reporting Items for Systematic Reviews and Meta-Analysis (PRISMA) [15] guidelines was conducted between April and June 2021. A draft list of key terms and truncations were established focusing on broad terms related to mental health, negative emotional symptoms, psychological wellbeing and COVID-19. The final combination of keywords included: coronavirus, COVID-19, SARS-COV-2, lockdown, negative emotion, mental health, anxiety, depression, stress, European, students. Studies were included if they were focused on European countries, were cross-sectional with their time period was during the lockdown period of the COVID-19 outbreak, the full text was available, and they focused on negative emotional symptoms of university students. Studies with insufficient data information or incompatible data (i.e., such as only reporting means) were excluded, along with studies that focus on school children. Further, there are several qualitative studies that were not included in this review and meta-analysis.

### 2.2. Data Extraction

Data extraction was carried out by the first author and screened by title and abstract. Full texts were then evaluated against the eligibility criteria, with those not meeting the requirements excluded at this stage by the searching author and their exclusion confirmed by all other authors. The collected data variables were the article title, author’s name, year of publication, country, sample size, number of students suffering from anxiety, depression or stress and assessment method (questionnaires, scales, etc.). No difference was made between the levels of condition but between experiencing or not negative emotional symptoms. The ‘no’ (not suffering) section ranged from ‘none’ to ‘normal’ levels of condition and the ‘yes’ (suffering) group ranged from ‘mild’ to ‘extremely severe’.

### 2.3. Statistical Analysis

Using the random-effects model [16], a meta-analysis was performed with the MetaXL, an add-in for meta-analysis in Microsoft Excel (Version 5.3, EpiGear) [17]. The double arcsine transformation of prevalence was used to avoid false representations of study weights and variance instability [18]. Included in the random effects pooling method, Cochran’s Q was used to test for heterogeneity [16]. However, Cochran’s Q test has low power when the number of studies is small in a meta-analysis [19]. Therefore, to quantify heterogeneity, I² and 95% confidence interval was used where I² values in the range 25–50% represented low, 50–75% moderate and 75–100% high heterogeneity [20]. A subgroup analysis on the different questionnaires was performed to explore the prevalence differences between questionnaires. A sensitivity analysis was conducted in order to determine the individual influence of each study on the overall result by omitting studies one by one. To assess for publication bias, the DOI plot and the LFK index were used. Both tools are recent and improved to detect bias in a meta-analysis in which the DOI plot allows for better visual detection of asymmetries than the funnel plot and the LFK index outperforms the Egger’s *p* value when it comes to power and sensitivity [21].

## 3. Results

**Figure 1:**
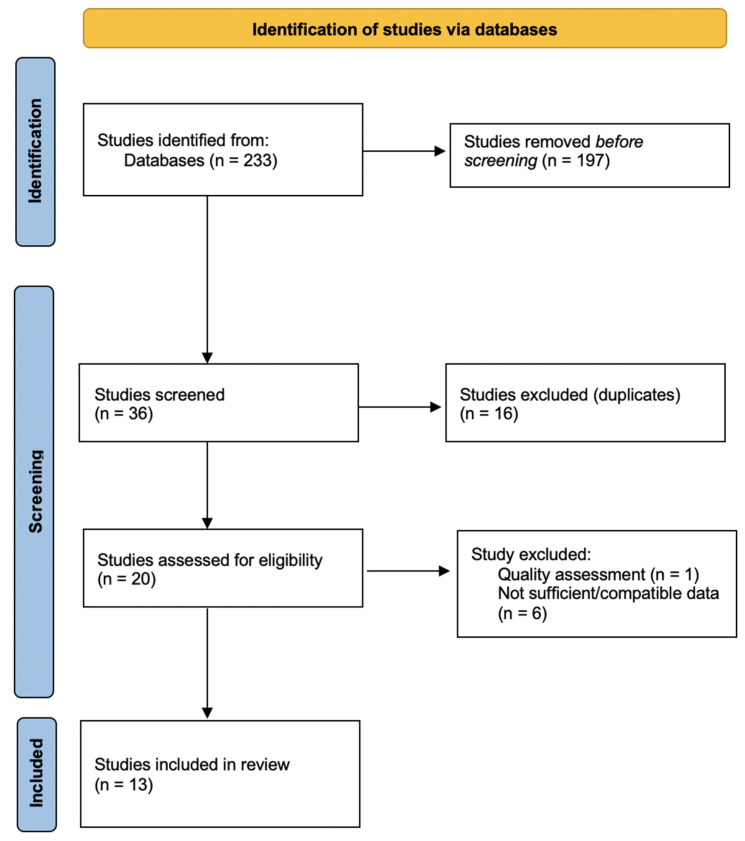
PRISMA Flowchart.

This meta-analysis regrouped 13 studies (Figure 1) with a total of 18,220 participants from nine European countries. The overall pooled prevalence for anxiety, depression and stress was 55% (95% CI: 45–64%), 63% (95% CI: 52–73%) and 62% (95% CI: 43–79%), respectively. Subgroup analysis showed a lower pooled prevalence for studies using the GAD-7 questionnaire (57% (95% CI: 44–69%) vs. 51% (95% CI: 29–73%)), but a higher pooled prevalence for those who used the PHQ-9 questionnaire 69% (95% CI: 56–82%) vs. 48% (95% CI: 25–71%). Descriptive statistics are provided in Table 1.

### 3.1. Anxiety

**Figure 2:**
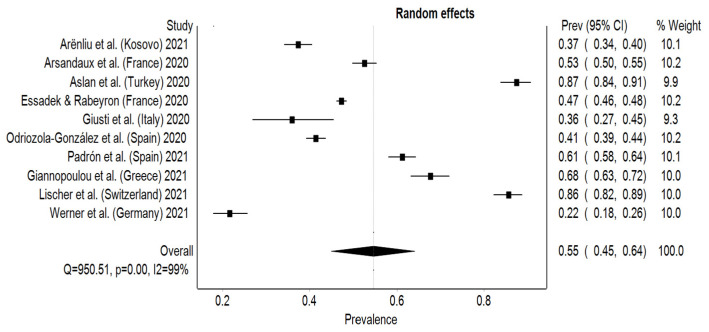
Forest plot of studies and their prevalence rates on anxiety.

**Figure 3:**
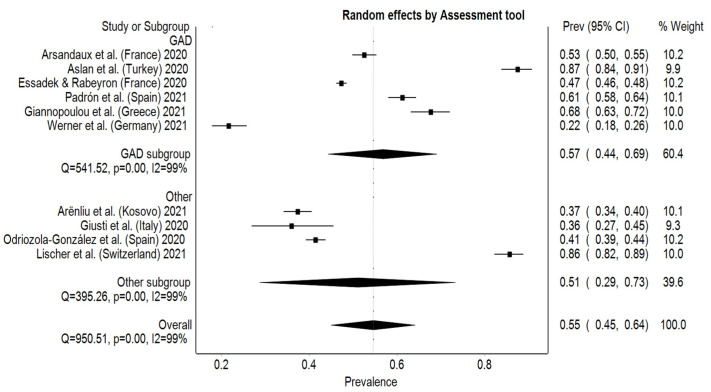
Subgroup analysis between GAD and other anxiety questionnaires.

The estimated overall pooled prevalence rate for anxiety was 55% (95% CI: 45–64%), with significant heterogeneity among studies (I² = 99%, *p* < 0.001). The prevalence rate ranged from 22% (95% CI: 18–26%) to 87% (95% CI: 84–91%), with Germany having the lowest prevalence rate for anxiety and Turkey the highest (Figure 2). In order to reject publication bias and ensure symmetry on the DOI plot, the LFK index has to fall inside the range of −1 to +1. In this study, there was a minor asymmetry with an LFK index of 1.36. Subgroup analysis on the assessment tool opposed the studies who used GAD-7 scale, which was the most frequent, to other questionnaires. The pooled prevalence for studies using GAD questionnaires was higher than compared to the other assessment tools with 57% (95% CI: 44–69%) and 51% (95% CI: 29–73%), respectively (Figure 3). Sensitivity test was performed manually by excluding each study one by one and recalculating for every exclusion. The pooled prevalence for anxiety ranged between 50% (95% CI: 42–59%) and 58% (95% CI: 49–68%) with the exclusion of Aslan et al. [24] and Werner et al. [33], respectively.

### 3.2. Depression

**Figure 4:**
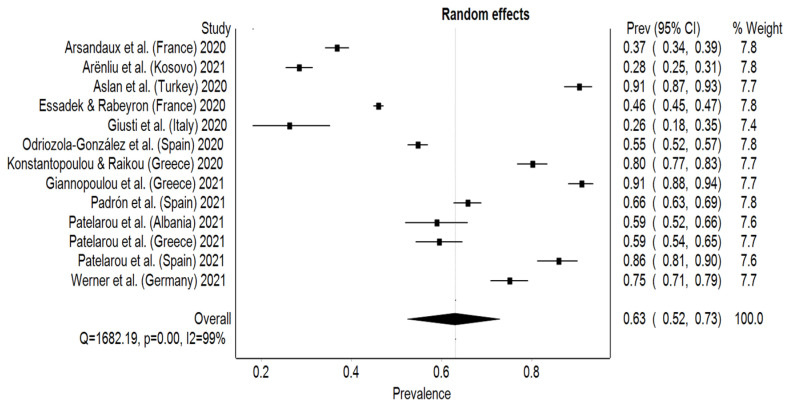
Forest plot of studies and their prevalence rates on depression.

**Figure 5:**
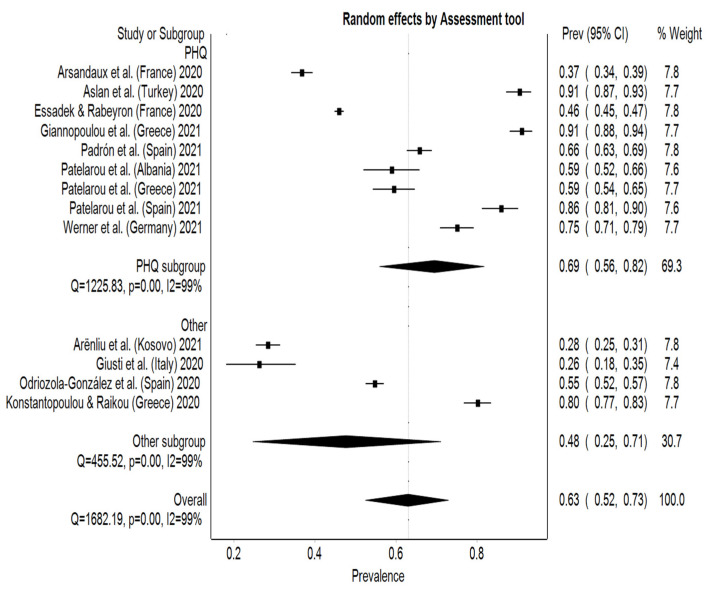
Subgroup analysis between PHQ and other depression questionnaires.

For depression, the estimated overall pooled prevalence rate was 63% (95% CI: 52–73%) with significant heterogeneity between studies (I² = 99%, *p* < 0.001). Italy reported the lowest prevalence rate of 26% (95% CI: 18–35%), while Greek and Turkish students reported a prevalence rate of 91% (95% CI: 88–94%) and 91% (95% CI: 87–93%), respectively [24,26,27] (Figure 4). An asymmetry can be seen on the DOI plot, which the LFK index confirmed as being a major asymmetry (LFK index = 3.9). Subgroup analysis on the assessment tools compared the overall pooled prevalence results from studies using the PHQ to the rest of the studies. Seven studies used the PHQ-9 obtaining a higher pooled prevalence rate, 69% (95% CI: 56–82%), than the other four studies that used other questionnaires, 48% (95% CI: 25–71%) (Figure 5). The range for the pooled prevalence in the sensitivity test was between 60% (95% CI: 50–70%), while excluding Aslan et al. [24] or Giannopoulou et al. [26], and 66% (95% CI: 55–76%), with the exclusion of Gisuti et al. [27] or Arënliu et al. [22].

### 3.3. Stress

**Figure 6:**
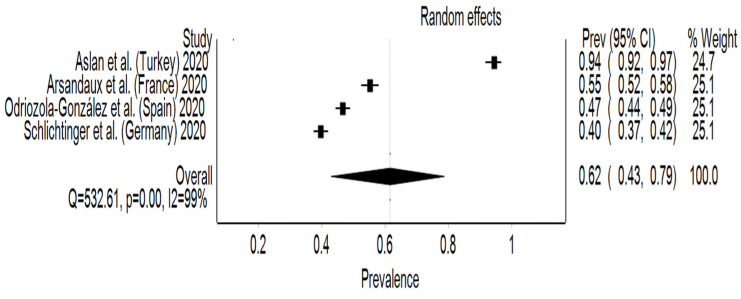
Forest plot of studies and their prevalence rates on stress.

The estimated overall pooled prevalence rate for stress was 62% (95% CI: 43–79%), with a significant heterogeneity between studies (I² = 99%, *p* < 0.001). Germany reported the lowest prevalence rate, 40% (95% CI: 37–42%), while Turkey had the highest stress prevalence rate with 94% (95% CI: 92–97%) (Figure 6). The DOI plot revealed a major asymmetry with an LFK index of 2.76. The sensitivity test reported a pooled prevalence rate of 47% (95% CI: 39–56%), when excluding Aslan et al. [24], and 69% (95% CI: 43–91%) when excluding Schlichtinger et al. [32].

## 4. Discussion

The aim of this study was to analyze the impact of the COVID-19 pandemic on students’ MH in Europe during the lockdown period. A decrease in mental wellbeing and an increased risk of developing mental disorders have been linked to pandemics in general [34]. Recent literature exposed high prevalence rates of some psychological aspects such as anxiety, depression, stress and distress in several countries around the world in the general population [9,10]. Therefore, through the lockdown situation and the switch to online classes, an increase and development of negative emotional symptoms was expected in the student population.

The COVID-19 pandemic brought devastating consequences, not only economically but also psychologically, especially in the younger population. Pre-pandemic, Kessler et al. [35] reported that 6.6% (95% CI: 5.9–7.3%) of the general population of the USA suffered from a major depressive disorder in 2001. Johansson et al. [36] reported a pooled prevalence of 10.8% (95% CI: 9.1–12.5%) for depression, 14.7% (95% CI: 12.7–16.6%) for anxiety and 8.3% (95% CI: 6.8–9.9%) for both in the Swedish population. The European Study of the Epidemiology of Mental Disorders project (ESEMeD) [37] reported that 1 person in 10 of the general population of six European countries (Belgium, France, Germany, Italy, Netherlands, Spain) experienced a mental disorder during the 12 months prior. The pooled prevalence for any anxiety, mental or mood disorder was 6.4% (95% CI: 6.0–6.8%), 9.6% (95% CI: 9.1–10.1%) and 4.2% (95% CI: 3.8–4.6%), respectively, while the prevalence for major depression was 3.9% (95% CI: 3.6–4.2%). In comparison to 2004, the prevalence rates increased drastically in 2021 during the lockdown period of the COVID-19 global pandemic.

Concerning anxiety symptoms, 9 of the 13 studies identified in this meta-analysis, reported an overall prevalence of 49.1%. Meaning, almost half of the participants experienced anxiety symptoms during lockdown. According to the meta-analysis of Wu et al. [38], the overall pooled prevalence of anxiety in students was 28.2% (95% CI: 16.8–41.2%), while Ma et al. [39] found 11% of Chinese students experienced anxiety symptoms. Furthermore, 45.4% of the 898 U.S. young adults reported high anxiety scores (GAD-7 scores ≥ 10) [40]. The result of this last study is close to the pooled prevalence of the current meta-analysis, but the Chinese students reported similar depression symptoms, but much lower prevalence rate for anxiety. For students in China, qualitative studies report this increase in anxiety is caused by a lack of social interaction, intensified by online studying and uncertainty of examination schedules [41], with similar findings in the USA [42] and the UK [43].

In this meta-analysis, 10 out of 13 studies reported an impact in depression, with the overall prevalence reaching 59.7%. Such high prevalence rates can also be observed in student population from other continents. A Chinese study with 746,217 students reported that 45% had probable clinical acute stress, anxiety or depression symptoms. The prevalence rate only for depression was 21.1% [39]. Wu et al. [38] reported in their systematic review and meta-analysis, regrouping 66 studies, that the overall pooled prevalence of depression in students was 34.8% (95% CI: 16.4–55.9%). A study on 898 U.S. young adults also reported high levels of depression (43.3%, PHQ-8 scores ≥ 10). The authors found a significant link between loneliness, COVID-19 specific worries, a low distress tolerance and high levels of depression [40]. Being isolated during a pandemic increases the probability of suffering from depressive symptoms [44]. This study supports the findings above and partly explains the increase of the prevalence of depression. Similarly, students in China have been recognized as having higher depressive levels during COVID-19 [45]. Increases in loneliness have also been reported in UK university students [43], with the authors acknowledging that many students are confined to small rooms with little or no outdoor space, leading to feelings of entrapment. With these results, it can be suggested that Europe has the highest prevalence rate of depression. This may be due to the severity of lockdown rules, the duration of the isolation period, support of the governments or other population/country related aspects.

Lastly, 4 of the 13 identified studies for this meta-analysis focused on the implication of stress with the overall prevalence reaching 47%. Not much less than the European students, Ma et al. [39] reported that acute stress was the most common problem in Chinese students with a prevalence rate of 34.9%. However, a study on 2059 students from an American university reported that 88% experienced moderate to severe stress, which is almost double that of European students and more than double when comparing with Chinese students [46]. Qualitative studies support this finding with increased stress caused by lockdowns during the pandemic described by the university [42]. This is suggested to have greatest impact on first-year students, who were already trying to adapt to commencing university life [43].

Moreover, in order to explain the high prevalence rates, the literature suggests that quarantines, reduced contact, reduced intimacy and social isolation are related to physical and mental health degradation [8,47]. These restrictions were present for weeks and sometimes months during the lockdown period, with long periods of isolation leading to a loss in connectedness and decreases in university students [43]. The results of this analysis demonstrate that the number of students developing negative emotional symptoms is increasing, possibly at the expense of mental wellbeing. This will have long-term implications, even after the pandemic, such as low quality of life, anxiety or depression disorders, post-traumatic stress disorder, low general health and cardiovascular diseases [48]. An increasing demand for psychological help from professionals, due to this increase in negative emotional symptoms, can be expected. Son et al. [42] called for urgent development of intervention and prevention strategies to support the mental health of university students.

Numerous variables can have a different impact on the situation. For example, students living with their families, having their support and the interaction with them, will most likely have a lower incidence of developing negative emotional symptoms compared to students living alone and away from their beloved ones. Similarly, to ensure for social distancing and minimizing contact, universities decided to switch to online classes. Aside from students feeling disadvantaged by remote learning [43], this decreases the opportunity to socialize with others, increasing the prevalence of anxiety and depression to a higher level compared to the general population under normal conditions [22,23,24,25,26,27,28,29,30,31,32,33].

### Limitations

The results of this study have to be interpreted carefully because some studies have very high prevalence rates, which influences the overall pooled prevalence. Likewise, some of the studies have a small sample size that often isn’t representative for the whole student population of that country. In order to regroup as much data as possible from different countries, no difference was made between the levels of condition but between experiencing (yes) or not experiencing (no) symptoms. Since the ‘yes’ data section regrouped the conditions from mild to severe or extremely severe, this partly explains the high prevalence rates. A high level of heterogeneity between studies and major asymmetry was found for all three symptoms which suggests a risk of publication bias. The low number of studies does not include the entire or most of the European continent. Furthermore, some studies of different countries had to be dropped because of their incompatible data, which limited the findings and representation of the situation.

## 5. Conclusions

The COVID-19 pandemic has caused considerable upheaval for university students. The amount of uncertainty regarding classes and examinations has placed increased stress on students, which has resulted in an amplified amount of negative emotional symptoms being experienced. This systematic review and meta-analysis has identified a significant increase in anxiety, depression and stress for university students across Europe. The long-term effect of this will need to be monitored, but governments, universities and other higher education providers should consider the mental health of students and provide strategies to support their psychological wellbeing.

## Figures and Tables

**Table 1:** Study Characteristics.

Author	Country	Sample Size (n)	Assessment Tools	Percentage of Anxiety	Percentage of Depression	Percentage of Stress
Arënliu et al. [22]	Kosovo	904	HADS	37.30%	28.30%	/
Arsandaux et al. [23]	France	1335	GAD-7/PHQ-9	52.50%	36.80%	55.10%
Aslan et al. [24]	Turkey	358	GAD-7/PHQ-8/PSS-10	87.43%	90.50%	94.41%
Essadek & Rabeyron [25]	France	8004	GAD-7/PHQ-9	47.30%	46%	/
Giannopoulou et al. [26]	Greece	452/435	GAD-7/PHQ-9	67.60%	91%	/
Giusti et al. [27]	Italy	103	SAS/BDI	35.90%	26.20%	/
Konstantopoulou & Raikou [28]	Greece	570	BDI	/	80.20%	/
Lischer et al. [29]	Switzerland	458	PHQ-4	85.60%	/	/
Odriozola-Gonzalez et al. [30]	Spain	1944	DASS-21	41.40%	54.70%	46.60%
Padrón et al. [3]	Spain	932	GAD-7/PHQ-9	61.20%	65.80%	/
Patelarou et al. [31]	Albania	197	PHQ-9	/	58.90%	/
	Greece	348	PHQ-9	/	59.50%	/
	Spain	242	PHQ-9	/	86%	/
Schlichtinger et al. [32]	Germany	1943	Self-reported questionnaire	/	/	39.60%
Werner et al. [33]	Germany	430	GAD-2/PHQ-9	21.60%	75.10%	/

HADS = Hospital Anxiety Depression Scale; GAD = General Anxiety Disorder; PHQ = Patient Health Questionnaire; PSS = Perceived Stress Scale; SAS = Self-rating Anxiety Scale; BDI = Beck’s Depression Inventory; DASS = Depression Anxiety Stress Scales.

## Data Availability

Not applicable.
